# Investigation of Influenza A(H5N1) Virus Neutralization by Quadrivalent Seasonal Vaccines, United Kingdom, 2021–2024

**DOI:** 10.3201/eid3106.241796

**Published:** 2025-06

**Authors:** Phoebe Stevenson-Leggett, Lorin Adams, David Greenwood, Abi Lofts, Vincenzo Libri, Bryan Williams, Sonia Gandhi, Charles Swanton, Steve Gamblin, Edward J. Carr, Ruth Harvey, Nicola S. Lewis, Mary Y. Wu, Emma C. Wall, Crick Neutralization Consortium, Legacy Investigators

**Affiliations:** The Francis Crick Institute, London, UK (P. Stevenson-Leggett, L. Adams, D. Greenwood, A. Lofts, S. Gandhi, C. Swanton, S. Gamblin, E.J. Carr, R. Harvey, N.S. Lewis, M.Y. Wu, E.C. Wall); Royal Veterinary College, London (L. Adams, N.S. Lewis); University College London Hospitals National Institute for Health Research Biomedical Research Unit, London (V. Libri, B. Williams, E.C. Wall); University College London, London (V. Libri, B. Williams, S. Gandhi, C. Swanton); University College London Centre for Kidney and Bladder Health, London (E.J. Carr); University College London Division of Infection and Immunity, London (E.C. Wall)

**Keywords:** influenza A, influenza, zoonoses, viruses, highly pathogenic avian influenza, neutralization, quadrivalent influenza vaccine, zoonotic transmission, United Kingdom

## Abstract

We tested cross-neutralization against highly pathogenic avian influenza A(H5N1) virus in adults vaccinated with 2021–2023 seasonal quadrivalent influenza vaccine in the United Kingdom. Seasonal quadrivalent influenza vaccines are unlikely to protect vulnerable persons against severe H5N1 disease during widespread transmission. Enhanced measures are needed to protect vulnerable people from H5N1 virus infection.

Highly pathogenic avian influenza (HPAI) A(H5N1) clade 2.3.4.4b viruses have been spreading worldwide among wild birds and poultry since 2020 (*1*). Officials in multiple US states confirmed spillover mammal infections in dairy cattle and detection in commercial milk samples in early 2024, enabled by uninterrupted cattle movement (*2*). Reports following documented human transmission in 2024 show evidence of H5N1 seroconversion in 7% of sampled dairy workers and that >30% of dairy herds in California are affected (*3*,*4*). Cow–human zoonotic transmission causing symptomatic but nonsevere infections have been reported in dairy workers, and 1 case was reported in September 2024 in a hospitalized person without exposure to animals and subsequent mild infection among healthcare workers caring for that patient (https://www.cdc.gov/bird-flu/spotlights/h5n1-response-09272024.html). However, a lack of widespread surveillance in cows and dairy and healthcare workers risks substantial underascertainment of bovine–human transmission events (*5*), which are further suggested by detection of H5N1 clade 2.3.4.4b viruses in Texas wastewater in March–July 2024 (*6*). In addition, fomite transmission through commercial milking equipment has been proposed as the primary route of cow–human transmission (N.J. Halwe et al., unpub. data, https://doi.org/10.1101/2024.08.09.607272). 

Although pasteurization renders HPAI H5N1 clade 2.3.4.4b viruses nonviable, viable virions have been detected in unpasteurized milk and caused fatal disease in cats that ingested the milk (*4*,*7*). Ongoing limited surveillance, detection, and containment of H5N1 clade 2.3.4.4b virus in the United States in both animal and human populations has led to mounting concern that this virus is gaining adaptions that could lead to effective human–human transmission, which could be catastrophic for clinically vulnerable people, particularly those who are immunocompromised and at the extremes of age (*2*).

Candidate vaccine viruses for pandemic preparedness have been recommended since the reemergence of A/goose/Guangdong/1/1996 lineage influenza A(H5) viruses in 2003. Vaccines against H5 clades, including clade 2.3.4.4b, have been in development, and some countries have small stockpiles. Whether those vaccines protect against severe disease is unknown, and current supplies to combat epidemic or pandemic spread of H5 virus are limited (*8*). 

Adults vaccinated with licensed H5N1 vaccines generate cross-reactive neutralizing antibodies against clade 2.3.4.4b viruses (*9*), but less is known about cross-protection against H5N1 viruses from quadrivalent influenza vaccines (QIVs). Antigen-specific B-cell monoclonal neutralizing antibodies (nAbs) against H5N1 virus can be found after QIV in humans (*10*), and studies in ferrets show further nAbs generated by seasonal influenza exposure might offer some protection against HPAI H5 challenge (S. Lakdawala et al., unpub. data, https://doi.org/10.21203/rs.3.rs-4935162/v1); however, QIV vaccination did not protect against H5N1 virus in mice (*11*). We investigated whether vaccine-induced immunity generated by seasonal QIVs would partially boost cross-reactive immunity against HPAI H5N1 virus in humans.

## The Study

We compared humoral neutralization of 2 H5N1 viruses, A/dairy_cattle/Texas/24-008749-002/2024 (2.3.4.4b) and A/Cambodia/NPH230776/2023 (2.3.2.1c), in serum samples alongside a seasonal influenza A(H1N1) virus isolate, A/Wisconsin/67/2022, before and after QIV in participants from the University College London Hospitals–Francis Crick Institute Legacy study cohort (https://clinicaltrials.gov/study/NCT04750356) (Appendix Figure). The study included 61 adults (median age 49 [range 38–58] years); 44 (72%) were women, and 17 (28%) were men. Thirty (49%) adults were vaccinated in only the 2021–22 season, but all 61 were vaccinated in >1 study season (2021–22, 2022–23, and 2023–24). Median sampling duration after vaccine dose for 2021–22 was 81 (interquartile range [IQR] 61–81) days, for 2022–23 was 67 (IQR 38–68) days, and for 2023–24 was 77 (IQR 44–77) days. Twenty-seven (44%) participants reported a single underlying condition (Table).

**Table:** Characteristics of patients in investigation of influenza A(H5N1) virus neutralization by quadrivalent seasonal vaccines, United Kingdom, 2021–2024*

Characteristic	Sampling season		
	2021–22, n = 30	2022–23, n = 54	2023–24, n = 59
Sex			
F	21 (70)	40 (74)	43 (73)
M	9 (30)	14 (26)	16 (27)
Median age, y (IQR)	56 (40–62)	49 (38–58)	49 (38–58)
Sampling site			
Crick	19 (63)	31 (57)	34 (58)
NHS sites UCLH or CNWL	11 (37)	23 (42)	25 (42)
Underlying conditions, any	16 (53)	24 (44)	25 (42)
Type 1 diabetes	1 (3.3)	1 (1.9)	1 (1.7)
Type 2 diabetes	2 (6.7)	2 (3.7)	2 (3.4)
Cancer, stroke, or heart problems	2 (6.7)	3 (5.6)	4 (6.8)
High blood pressure	5 (17)	5 (9.3)	6 (10)
Asthma or COPD	3 (10)	6 (11)	7 (12)
Alcohol consumption			
Never	0 (0)	4 (10)	5 (11)
Monthly or less	6 (26)	12 (30)	13 (30)
2–4 times/mo	0 (0)	0 (0)	0 (0)
2 or 3 times/wk	11 (48)	19 (48)	21 (48)
>4 times/wk	6 (26)	5 (13)	5 (11)
Unknown	7	14	15
Smoking status			
Never smoker	25 (83)	41 (76)	47 (80)
Ex-smoker	5 (17)	10 (19)	9 (15)
Current smoker	0 (0)	3 (5.6)	3 (5.1)
Median days from vaccine to sample collection (IQR)	82 (61–81.5)	67 (33.75–67)	61 (26–61)

*Values are no. (%) except as indicated. CNWL, Central Northwest London; COPD, chronic obstructive pulmonary disease; IQR, interquartile range; NHS, National Health Service; UCLH, University College London Hospitals.

In line with their effectiveness against seasonal influenza, each QIV generated a statistically significant boost in serum neutralization of A/Wisconsin/67/2022 in each season tested (p = 0.003–0.007 by χ^2^ test) (Figure). In contrast, HPAI H5 virus neutralization in our cohort of healthy adults was blunted or absent. In prevaccine serum samples, we detected neutralization above background against A/Cambodia/NPH230776/2023 in a few samples but did not detect any neutralization against A/dairy_cattle/Texas/24-008749-002/2024 isolate (Figure). No seasonal QIV resulted in a cross-neutralization boost against either HPAI H5 virus (Figure).

**Figure:**
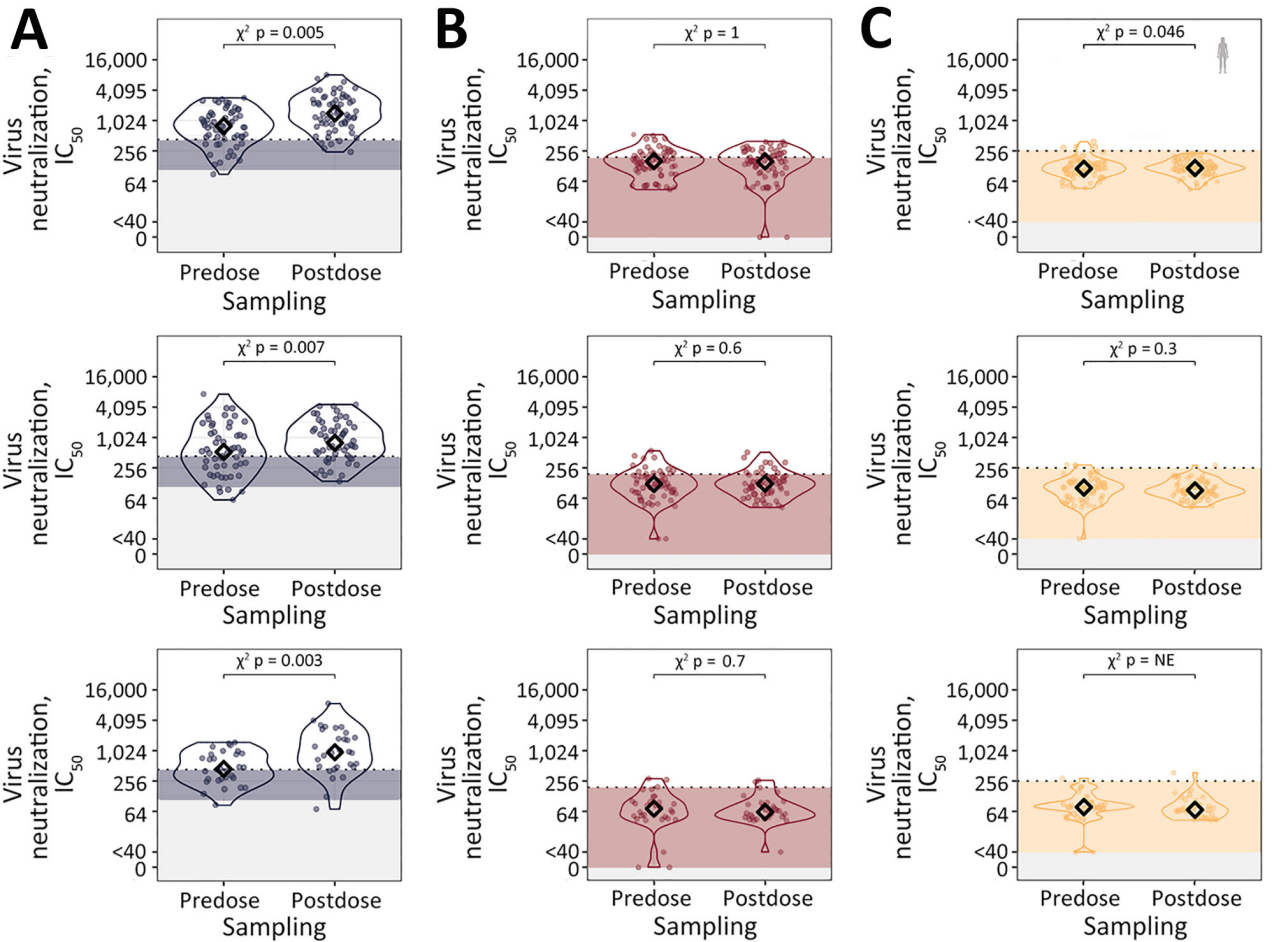
Live-virus neutralization of preseasonal and postseasonal quadrivalent influenza vaccination in a study of influenza A(H5N1) virus neutralization by quadrivalent seasonal vaccines, United Kingdom, 2021–2024. Violin plots show paired serum samples before and after each seasonal vaccine (top row, 2023–24, n = 59; middle row, 2022–23, n = 54; bottom row, 2021–22, n = 30) tested against pandemic H1 and 2 highly pathogenic avian influenza H5 strains: H1N1 strain (A), H5N1 Cambodia strain (B), and H5H1 Texas dairy strain (C). Virus neutralization reported as log_2_-transformed IC_50_. Black diamonds indicate median IC_50_. Nonspecific cross-reactivity levels are shown as shaded areas. p values from McNemar tests are shown, comparing the number of persons above background (dashed line) before and after quadrivalent influenza vaccination. IC_50_, reciprocal titer at which 50% of viral infection is inhibited; NE, not estimated.

Ongoing adaptation of HPAI H5N1 clade 2.3.4.4b virus in cows and other mammal hosts found on dairy farms, including rodents and cats, substantially increases the risk for a major HPAI H5N1 virus epidemic or pandemic in humans (*2*,*4*). The paucity of human serologic memory against either H5N1 virus strain raises the potential for widespread vulnerability to infection within the adult population. We observed a predictable boost to neutralizing titers against the contemporary seasonal influenza A(H1N1) virus (A/Wisconsin/67/2022) that was absent for the 2 clinically relevant H5N1 viruses tested in our high-throughput neutralization assay (Appendix). Neutralizing antibody titers have long been used as a correlate of protection against seasonal influenza (*12*); thus, our observations suggest seasonal QIVs are unlikely to offer adequate serologic protection against H5N1 virus.

Immunity against influenza evolves throughout the lifespan, and early infection exposures influence subsequent antibody responses after infection and vaccination (*13*). Few participants in our study had detectable neutralizing titers above background to the 2.3.2.1c A/Cambodia/NPH230776/2023 virus and none to the 2.3.4.4b A/dairy_cattle/Texas/24-008749-002/2024 virus. Together with our observed lack of QIV boosting, our results suggest that strategies reliant on existing population-level or QIV-based immunity against H5N1 virus infection must be approached with caution.

One limitation of this study is the lack of in vivo challenge to test for cross-protection. Some studies have reported transient protection against H5N1 challenge after transferring QIV-vaccinated human serum to mice, which was not accurately predicted by in vitro assays, including virus neutralization assays (*14**,**15*). Cross-neutralization might also occur in the absence of nAbs, but without in vivo testing, we cannot conclusively determine the extent to which the QIV might provide protection against H5N1 virus. A second limitation is that our cohort, predominately working age, healthy adults receiving occupational QIV, do not represent a population at high risk for severe influenza disease and death. However, they represent an immunocompetent population and would be expected to have the most robust detectable immunity. Third, although we tested 2 H5N1 viruses associated with recent human disease, despite extensive efforts, we could not eliminate background signal in our assay. Thus, we were unable to fully quantify neutralization at lower titers and opted to describe the range within which we detected background signal. As of 2025, no neutralization titers were available from postinfection serum samples in dairy farm workers to further refine that cutoff (*6*). Further investigation is required to address the issue of background signal. However, other studies suggest nonspecific inhibition by human serum as a possible explanation for low-level readouts for protection (*15*). Finally, our use of whole virus to assess nAb titers did not allow determination of the extent to which hemagglutinin- or neuraminidase-specific antibodies might have contributed to overall neutralization. However, the high-throughput live-virus neutralization we describe (Appendix) is a highly valuable tool for pandemic preparedness, offering a method for near real-time analysis of serum-based immunity to emerging viruses in large cohorts.

## Conclusions

The effectiveness of QIV against influenza A(H5N1) virus remains uncertain, and clarification on the extent of cross-protection in humans is urgently needed. Considering that uncertainty, timely and effective deployment of targeted vaccines would be crucial during widespread influenza A(H5N1) outbreaks. To reduce risks for severe illness and death requires enhanced measures that mitigate the spread of HPAI H5N1 viruses to humans, accelerated pipelines for H5-directed influenza vaccines, and systems that rapidly and equitably reach clinically vulnerable persons worldwide (*2*).

Appendix Additional information for investigation of influenza A(H5N1) virus neutralization by quadrivalent seasonal vaccines, United Kingdom, 2021–2024.

## References

[1] Peacock TP, Moncla L, Dudas G, VanInsberghe D, Sukhova K, Lloyd-Smith JO, et al. The global H5N1 influenza panzootic in mammals. Nature. 2025;637:304–13. 10.1038/s41586-024-08054-z 39317240

[2] Lewis N, Beer M. Stop H5N1 influenza in US cattle now. Science. 2024;385:123. 10.1126/science.adr5866 38991057

[3] Mellis AM, Coyle J, Marshall KE, Frutos AM, Singleton J, Drehoff C, et al. Serologic evidence of recent infection with highly pathogenic avian influenza A(H5) virus among dairy workers—Michigan and Colorado, June–August 2024. MMWR Morb Mortal Wkly Rep. 2024;73:1004–9. 10.15585/mmwr.mm7344a3 39509348 PMC11542770

[4] Burrough ER, Magstadt DR, Petersen B, Timmermans SJ, Gauger PC, Zhang J, et al. Highly pathogenic avian influenza A(H5N1) clade 2.3.4.4b virus infection in domestic dairy cattle and cats, United States, 2024. Emerg Infect Dis. 2024;30:1335–43. 10.3201/eid3007.240508 38683888 PMC11210653

[5] Shittu I, Silva D, Oguzie JU, Marushchak LV, Olinger GG, Lednicky JA, et al. A One Health investigation into H5N1 avian influenza virus epizootics on two dairy farms. Clin Infect Dis. 2025;80:331–8. 10.1093/cid/ciae576 39658318

[6] Tisza MJ, Hanson BM, Clark JR, Wang L, Payne K, Ross MC, et al. Sequencing-based detection of avian influenza A(H5N1) virus in wastewater in ten cities. N Engl J Med. 2024;391:1157–9. 10.1056/NEJMc2405937 39259887 PMC12435930

[7] Guan L, Eisfeld AJ, Pattinson D, Gu C, Biswas A, Maemura T, et al. Cow’s milk containing avian influenza A(H5N1) virus—heat inactivation and infectivity in mice. N Engl J Med. 2024;391:87–90. 10.1056/NEJMc2405495 38785313 PMC12809964

[8] World Health Organization. Zoonotic influenza: candidate vaccine viruses and potency testing reagents [cited 2024 Oct 5]. https://www.who.int/teams/global-influenza-programme/vaccines/who-recommendations/zoonotic-influenza-viruses-and-candidate-vaccine-viruses

[9] Khurana S, King LR, Manischewitz J, Posadas O, Mishra AK, Liu D, et al. Licensed H5N1 vaccines generate cross-neutralizing antibodies against highly pathogenic H5N1 clade 2.3.4.4b influenza virus. Nat Med. 2024;30:2771–6. 10.1038/s41591-024-03189-y 39013430

[10] Throsby M, van den Brink E, Jongeneelen M, Poon LL, Alard P, Cornelissen L, et al. Heterosubtypic neutralizing monoclonal antibodies cross-protective against H5N1 and H1N1 recovered from human IgM+ memory B cells. PLoS One. 2008;3:e3942. 10.1371/journal.pone.0003942 19079604 PMC2596486

[11] Beukenhorst AL, Frallicciardi J, Rice KL, Koldijk MH, Moreira de Mello JC, Klap JM, et al. A pan-influenza monoclonal antibody neutralizes H5 strains and prophylactically protects through intranasal administration. Sci Rep. 2024;14:3818. 10.1038/s41598-024-53049-5 38360813 PMC10869794

[12] Verschoor CP, Singh P, Russell ML, Bowdish DM, Brewer A, Cyr L, et al. Microneutralization assay titres correlate with protection against seasonal influenza H1N1 and H3N2 in children. PLoS One. 2015;10:e0131531. 10.1371/journal.pone.0131531 26107625 PMC4479562

[13] Edler P, Schwab LSU, Aban M, Wille M, Spirason N, Deng YM, et al. Immune imprinting in early life shapes cross-reactivity to influenza B virus haemagglutinin. Nat Microbiol. 2024;9:2073–83. 10.1038/s41564-024-01732-8 38890491

[14] Roos A, Roozendaal R, Theeuwsen J, Riahi S, Vaneman J, Tolboom J, et al. Protection against H5N1 by multiple immunizations with seasonal influenza vaccine in mice is correlated with H5 cross-reactive antibodies. Vaccine. 2015;33:1739–47. 10.1016/j.vaccine.2015.01.070 25659276

[15] Roozendaal R, Tolboom J, Roos A, Riahi S, Theeuwsen J, Bujny MV, et al. Transient humoral protection against H5N1 challenge after seasonal influenza vaccination of humans. PLoS One. 2014;9:e103550. 10.1371/journal.pone.0103550 25075622 PMC4116209

