# Supplementary material for: Investigation of Influenza A(H5N1) Virus Neutralization by Quadrivalent Seasonal Vaccines, United Kingdom, 2021–2024
Source: Emerg Infect Dis. 2025 Jun;31(6):1202–6. doi: 10.3201/eid3106.241796 (PMC12123933; doi:10.3201/eid3106.241796)
Supplement: Appendix — Additional information for investigation of influenza A(H5N1) virus neutralization by quadrivalent seasonal vaccines, United Kingdom, 2021–2024. [file 24-1796-Techapp-s1.pdf]

Article DOI: https://doi.org/10.3201/eid3106.241796

EID cannot ensure accessibility for supplementary materials supplied by authors. Readers who have difficulty accessing supplementary content should contact the authors for assistance.

Investigation of Influenza A(H5N1) Virus Neutralization by Quadrivalent Seasonal Vaccines, United Kingdom, 2021–2024

Appendix

Materials and Methods

Clinical Cohort

The Legacy study (NCT04750356) is a prospective observational cohort, established in January 2021. Extensive descriptions of the cohort can be found in our prior reports (*1*,*2*). Legacy participants are offered seasonal influenza vaccination annually through the NHS/Crick employer access program, most recently in parallel with updated COVID-19 vaccinations. Legacy participants were included in this study if they reported receipt of a seasonal QIV over at least 2 winter seasons in the past 3 years, with blood drawn both within 90 days after the reported vaccination date and with a prevaccination blood draw collected at least 6 months after the preceding year's vaccination (Appendix Table). At each draw, serum was extracted and stored at −80°C for live-virus microneutralization assays as described below.

We adapted our whole-virus, high-throughput microneutralization assay for HPAI, including screening for background signal using naive ferret serum samples, antigenically irrelevant ferret antiserum, and recombinant human albumin, and found that albumin alone returned low but detectable titers across all strains (L. Adams et al., unpub. data, https://doi.org/10.1101/2024.09.13.24313549).

Virus Variants and Culture

Viruses were acquired from submitting laboratories through the Global Influenza Surveillance and Response System (*3*). A/Wisconsin/67/2022 (H1N1) was diluted in DMEM containing 1% penicillin/streptomycin, 25 mM HEPES, and 2.5 µg TPCK-trypsin, and passaged

in MDCK cells for 48 hours at 34°C, 5% CO_2. Influenza A(H5N1) viruses were propagated in the allantoic cavity of 10-day-old embryonated hen eggs at 35°C, 70% humidity, for 16–20 hours and briefly clarified by centrifugation according to standard methods (*4*).

In a recent study of 60 healthy volunteers, stratified by birth cohort, detectable cross-neutralization to HPAI (H5N1) was found in 8 of 23 (34%) older adults (born between 1940 and 1969) and absent in those born after 1970 (S. Lakdawala et al., unpub. data, https://doi.org/10.21203/rs.3.rs-4935162/v1).

High-Throughput Live-Virus Microneutralization Assay for Serum Samples

High-throughput live-virus microneutralization assays were performed for influenza by adapting our existing approach for SARS-CoV-2 neutralization (*1*,*2*,*5*,*6*; L. Adams et al., unpub. data, http://medrxiv.org/lookup/doi/10.1101/2024.09.13.24313549). MDCK-SIAT1 cells at 80% confluency were infected in a CL4 facility with selected influenza H5N1 isolates and CL3 facility for H1pdm in 384-well format in the presence of 10-fold serial dilutions of participant serum samples, diluted in DMEM containing 1% penicillin/streptomycin. After 6 (for H5N1 viruses) or 24 (H1pdm) hours of incubation at 37°C, cells were fixed using 4% formaldehyde (v/v), permeabilized with 0.2% TritonX-100 with 3% BSA in PBS (v/v), and stained for influenza nucleoprotein (NP) using Biotin-labeled-clone-2–8C antibody produced in-house in conjunction with an Alexa488-Streptavidin (Invitrogen S32354). Cellular DNA was stained using DAPI. Whole-well imaging at ×5 magnification was carried out using an Opera Phenix (Perkin Elmer) and fluorescent areas calculated using the Phenix-associated software Harmony (Perkin Elmer). Virus inhibition by patient serum samples was estimated from the measured area of infected cells/total area occupied by all cells in each well and then expressed as percentage of maximal (virus only control wells). Infected cells were identified by the presence of influenza NP staining. The inhibitory profile of each serum sample was estimated by fitting a 4-parameter dose response curve executed in SciPy. Neutralizing antibody titers are reported as the fold-dilution of serum required to inhibit 50% of viral replication (IC_{50}) and are further annotated if they lie above the quantitative range (>40,000), below the quantitative range (<40) but still within the qualitative range (i.e., partial inhibition is observed but a dose-response curve cannot be fitted because it does not sufficiently span the IC_{50}), or if they show no inhibition at all. To plot LV-N titers, serum samples with either no inhibition of viral entry, qualitative inhibition

below the quantitative range (40–40,000), or inhibition greater than the quantitative range were recoded as 5, 10, or 80,000, respectively.

Data Analysis, Statistics, and Availability

Data analysis was performed in R, using *ggplot2* for plotting (*7*), with PhyloPic silhouettes rendered with *rphylopic* (*8*). Nonspecific background cross-reactivity was defined as the minima and maxima IC_{50} across standardized irrelevant antiserum samples used in WHO Influenza collaborating centers (obtained from the Worldwide Influenza Centre at the Francis Crick Institute), and recombinant human albumin (New England Biolabs, B9200S–recombinant human albumin expressed in yeast, used at a starting concentration of 0.5 mg/mL). For example, for H5N1(dairy cattle/Texas), the minimum and maximum IC_{50} against H5N1 were measured across H3N2, flu B antiserum samples, and naive serum samples (Appendix Figure, panel A), and across fetal bovine serum samples, further non-H5N1 viruses and recombinant albumin (Appendix Figure, panel B). All these conditions contain albumin (of differing species), and some have irrelevant antibody (either against irrelevant influenza viruses or tonically produced IgG). We used the maxima and minima IC_{50} across these conditions to determine these albumin-defined backgrounds. These will be referred to as the albumin-defined backgrounds.

The albumin-defined backgrounds were H1pdm 233379 A/Wisconsin/67/2022: 105 0.0–432.3; A/Cambodia/NPH230776/2023: 5.0–192.4; A/dairy_cattle/Texas/24–008749–002/2024: 10.0–254.9.

Neutralization capacity in human serum samples was dichotomized into titers below and above the upper bounds of the albumin-defined backgrounds. McNemar's χ^2 tests for paired categorical data were applied to assess the significance of differences in neutralization before and after seasonal vaccination (*9*). Statistical tests were conducted with the *rstatix* R package (*10*).

References

1. Wall EC, Wu M, Harvey R, Kelly G, Warchal S, Sawyer C, et al. Neutralising antibody activity against SARS-CoV-2 VOCs B.1.617.2 and B.1.351 by BNT162b2 vaccination. Lancet. 2021;397:2331–3. PubMed https://doi.org/10.1016/S0140-6736(21)01290-3

2. Wall EC, Wu M, Harvey R, Kelly G, Warchal S, Sawyer C, et al. AZD1222-induced neutralising antibody activity against SARS-CoV-2 Delta VOC. Lancet. 2021;398:207–9. PubMed https://doi.org/10.1016/S0140-6736(21)01462-8

3. World Health Organization. Global influenza surveillance and response system [cited 2024 Oct 5]. https://www.who.int/initiatives/global-influenza-surveillance-and-response-system

4. World Organization for Animal Health. Chapter 3.3.4. Avian influenza (including infection with high pathogenicity avian influenza viruses) [cited 2024 Oct 19]. https://www.woah.org/fileadmin/Home/eng/Health_standards/tahm/3.03.04_AI.pdf

5. Carr EJ, Wu MY, Gahir J, Harvey R, Townsley H, Bailey C, et al.; Crick COVID Serology Consortium. Neutralising immunity to omicron sublineages BQ.1.1, XBB, and XBB.1.5 in healthy adults is boosted by bivalent BA.1-containing mRNA vaccination and previous Omicron infection. Lancet Infect Dis. 2023;23:781–4. PubMed https://doi.org/10.1016/S1473-3099(23)00289-X

6. Shawe-Taylor M, Greenwood D, Hobbs A, Dowgier G, Penn R, Sanderson T, et al.; Crick COVID Serology Pipeline Investigators, Legacy Investigators. Divergent performance of vaccines in the UK autumn 2023 COVID-19 booster campaign. Lancet. 2024;403:1133–6. PubMed https://doi.org/10.1016/S0140-6736(24)00316-7

7. Wickham H. ggplot2: elegant graphics for data analysis, 2nd ed. Cham (Switzerland): Springer; 2016.

8. Gearty W, Jones LA. rphylopic: an R package for fetching, transforming, and visualising PhyloPic silhouettes. Methods Ecol Evol. 2023;14:2700–8. https://doi.org/10.1111/2041-210X.14221

9. McNemar Q. Note on the sampling error of the difference between correlated proportions or percentages. Psychometrika. 1947;12:153–7. PubMed https://doi.org/10.1007/BF02295996

10. Kassambara A. rstatix: pipe-friendly framework for basic statistical tests [cited 2024 Sep 20]. https://cran.r-project.org/web/packages/rstatix/index.html

Appendix Table. Influenza strains tested in microneutralization assay: 233379 A/H1pdm/Wisconsin/67/2022, A/Cambodia/NPH230776/2023, and A/dairy_cattle/Texas24–008749–002/2024

Appendix Figure 1, panel B: influenza strain abbreviation	Full influenza strain
A/Cambodia/H5N1	A/Cambodia/NPH230032/2023(H5N1)
A/Guangdong/H1pdm	A/Guangdong/Maoan/SWL1536/2019 H1pdm
A/Cambodia/H3N2	A/Cambodia/e0826360/2020 H3N2
B/BVIC	B/Washington/02/2019/BVIC
A/Wisconsin/H1pdm	A/Wisconsin/588/2019/H1pdm
A/Victoria/H1pdm	A/Victoria/4897/2022 H1pdm
A/Swine/H1N2	A/Swine/Northern Ireland/2073-052022 H1N2
A/Ohio/H1N1v	IDCDC-RG48A A/Ohio/9/2015 H1N1v
A/Michigan/H1N2	IDCDC-RG58A A/Michigan/383/2018 H1N2
A/Hong Kong/H3N2	A/Hong Kong/45/2019 H3N2
A/Stockholm H3	A/Stockholm/5/2021 H3
B/Netherlands	B/Netherlands/10335/2023 BV

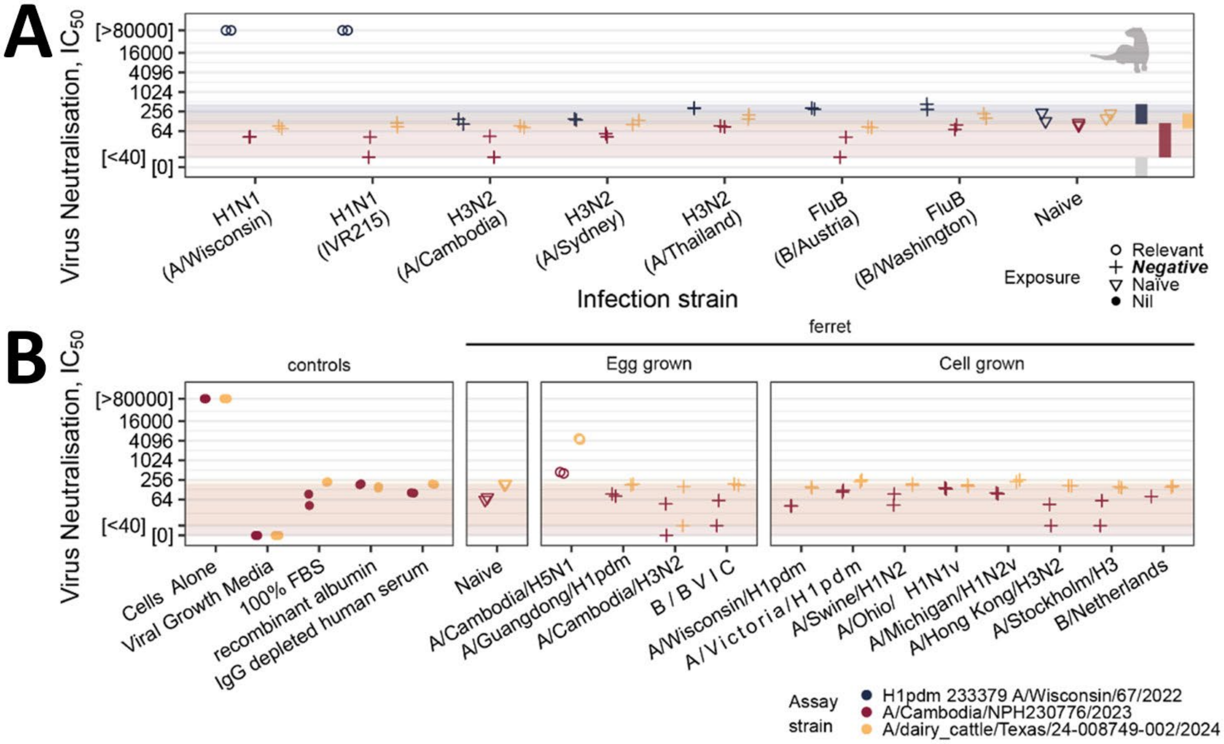

Appendix Figure. No cross-reactive boost in HPAI A(H5N1) neutralization in H1N1-infected ferret serum samples. A, B) Live-virus neutralization of influenza H1 and HPAI A(H5N1) from H1N1-exposed ferrets is plotted as log2-transformed IC_{50} (the reciprocal titer at which 50% of viral infection is inhibited). Different antiserum samples are shown along the *x*-axis, including influenza-naive control serum samples (triangles). Homologous and heterologous pairs of antiserum samples and assay strains are plotted as circles and pluses, respectively. Assay strains are indicated by color: H1N1 (blue), H5N1 Cambodia (red), and H5H1 Texas dairy (yellow). Background was defined as the minima and maxima IC_{50} across irrelevant antisera (see Methods) and is indicated by shading. A and B are presented separately because experiments were performed independently.
